# Conditioning of microvascular venous flaps in rats

**DOI:** 10.1038/s41598-023-28054-9

**Published:** 2023-01-19

**Authors:** Christian Heiliger, Lucas M. Ritschl, Andreas M. Fichter, Lukas K. Postl, Anastasios Kanatas, Klaus Dietrich Wolff, Thomas Mücke

**Affiliations:** 1 Department of Oral and Maxillofacial Surgery, School of Medicine, Technical University of Munich, Klinikum Rechts Der Isar, Munich, Germany; 2 Medical Faculty, Johannes Kepler University Linz, Linz, Austria; 3 St James Institute of Oncology and Leeds Dental Institute, Leeds Teaching Hospitals, Leeds, UK

**Keywords:** Preclinical research, Medical research, Head and neck cancer, Oral cancer, Medical imaging, Surgery, Reconstruction, Surgical oncology

## Abstract

Venous-only perfusion flaps have not been used widely because of the associated high failure rate. Tissue conditioning offers a broad scope of techniques that can be applied pre-, peri-, or postoperatively to promote the adaptation of the affected tissue to any subsequent stress. This study aimed to assess the survival rates associated with a pure venous perfusion flap and investigate whether the timing of the vascular conditioning can affect free flap survival. Forty-four rats were included in the experiment. Group I underwent veno-arterial anastomoses with epigastric graft with pure venous perfusion without tissue conditioning. Groups II and III were pretreated for 7 or 14 days with ischemic conditioning. These groups were compared with a control group (group IV) of conventionally perfused flaps. After the initial surgery, all flaps were assessed clinically, photometrically, and by indocyanine green videoangiography. The flap success rates were 0% in group I, 49.97% ± 24.34% in group II, and 64.95% ± 20.36% in group III. The control group showed an overall survival of 89.3% ± 6.51%. With suitable conditioning, pure venous blood supply can provide adequate perfusion in the rat epigastric flap model. The timing of vascular conditioning appears to be critical for flap survival.

## Introduction

Advancements in free tissue transfer have made a marked difference in the field of reconstructive microsurgery. Free flaps are the best reconstructive options after major head and neck surgery. However, the usual microvascular tissue transfer is not possible in rare situations^1^. Some of the challenges include recurrent diseases, a history of previous neck dissection, and previous radical or adjuvant radiotherapy to the neck. In some instances, surgery is the only available treatment modality, and the reduced availability or poor quality of neck vessels increased the complication rates^2–5^. In these patients, the neck vessels could affect the chance of cure, especially if surgery resulted in functional impairment^6^. In the absence of suitable arteries, venous perfusion could be an alternative for soft tissue reconstruction. Even though venous vessels are often the limiting factors in clinical practice, arterialized venous “flow-through” flaps could offer additional reconstruction option. Secondary venous perfusion via the arterial vessels of the flap could be also conceivable in the context of rescue operations. In the case of multivessel supply, the appropriate use of venous connecting vessels could also expand the surgical options.

Furthermore, an understanding of the underlying physiological changes in conditioning could result in further improvements in free flap transfer. Arterialized venous “flow-through” flaps have been used for the coverage of small soft tissue defects of the finger; however, pure venous flaps are difficult to predict, and lap failure or partial flap necrosis often occurs^7–12^. A purely venous perfusion approach has not shown satisfactory results in the past; as a result, various concepts have been described to improve the survival rate of venous flaps. These include surgical delay procedures^8,13^ or pre-arterialization of venous flaps^14,15^. Surgical delay is the predecessor of modern preconditioning techniques. Clinically, arterialized venous flaps have been used^7–11,16–21^; however, these have not yet been established for complex defect reconstruction^22,23^.

This study aimed to perfuse a flap with venous blood through the artery similar to arterialized venous flaps^8,9,19–21^ and explore flap survival with soft conditioning. In this study, the efferent vein was retrogradely pre-arterialized, and the flap was preconditioned with an ischemic delay procedure^8,13,24,25^ to ensure sufficient blood circulation of the tissue graft with venous blood after tissue transplantation.

## Materials and methods

A total of 51 male Wistar rats (280–320 g; Fa. Charles River; Kißlegg, Germany) were used in this study. All procedures were conducted in strict accordance with the recommendations and guidance for the care and use of laboratory animals of the National Institutes of Health, which received full approval by the local Ethical Committee on Animal Experimentation (Regierung von Oberbayern, AZ55.2–1-54–2532-129–10) by Technical University.

The study was carried out in compliance with the ARRIVE guideline.

Food and water were provided ad libitum. All surgical procedures were performed aseptically under intravenous general anesthesia composed of ketamine 100 mg/kg (Narketan^®^, Fa. Vétoquinol GmbH, Ravensburg, Germany) and xylazine 5 mg/kg (Rompun^®^, Fa. Bayer Vital GmbH, Leverkusen, Germany) as described elsewhere^26^. Every effort was made to minimize animal suffering.

### Surgical technique

The epigastric flap was raised as a pedicled flap based on the epigastric pedicle from the deep inferior vascular system with a standardized dimension of 4 × 7 cm, as comprehensively described elsewhere^27,28^. Surgery was performed with a microscope-type OPMI^®^ Pentero^®^ equipped with an integrated near-infrared videoangiography detection system and FLOW 800 tool (INFRARED 800, Carl Zeiss Meditec AG, Oberkochen, Germany) to subsequently analyze the fluorescence behavior over time and direction of blood flow after the anastomoses.

### Catheterization of the femoral vein

After the preparation of the right femoral vessels, the femoral vein was catheterized by longitudinal venotomy distal to the inguinal ligament. The catheter was secured by temporary vessel clips distally to the venotomy. Owing to repeated catheterizations, the vessel clips were removed, and the venotomy was closed with interrupted sutures after each operation.

### Veno-arterial anastomosis

**Figure 1:**
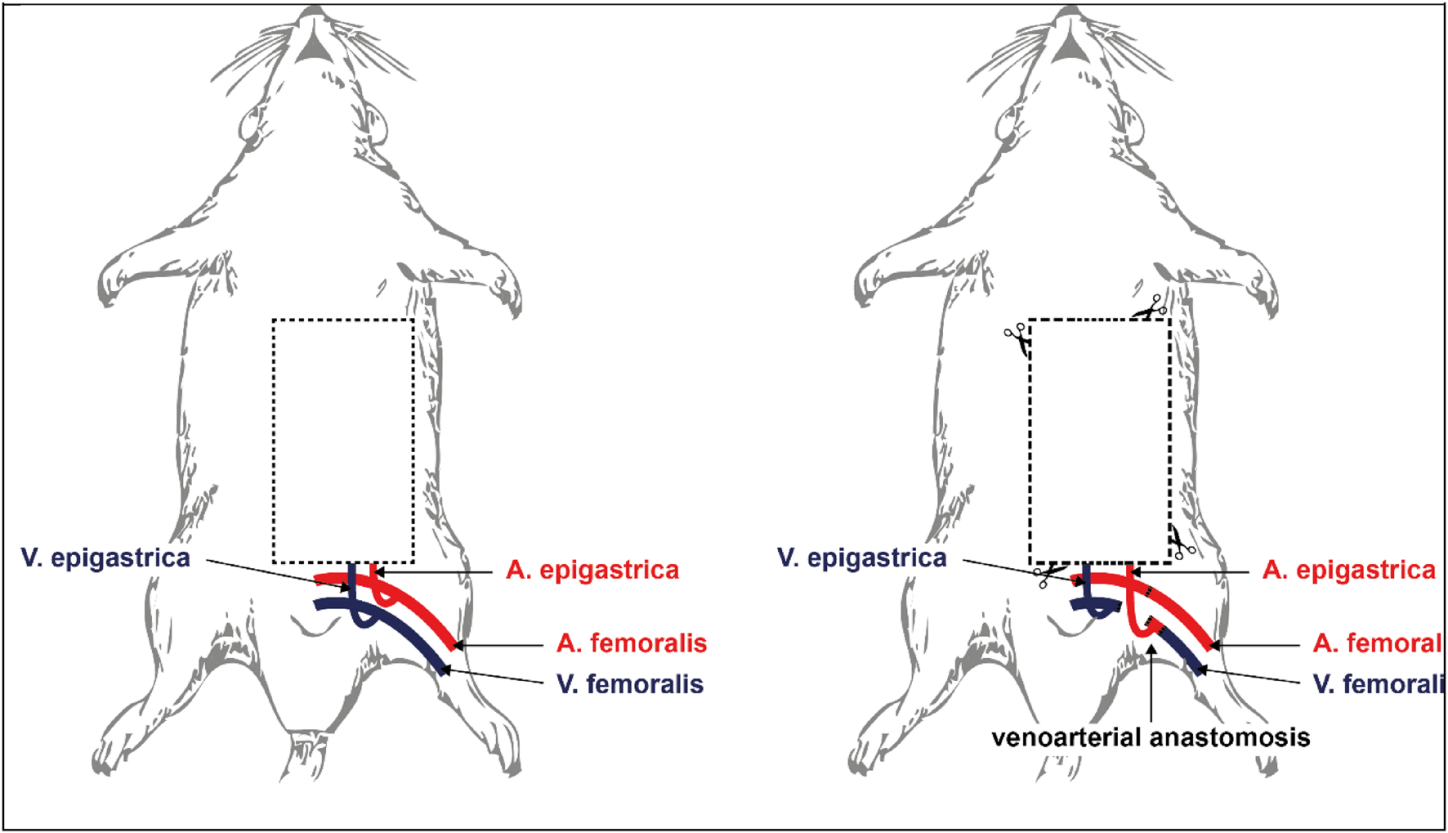
Schematic illustration of the experimental model. The femoral vein was ligated distal to the epigastric vein and the femoral artery proximal to the epigastric artery. The epigastric artery together with a distal part of the femoral artery was anastomosed with the distal femoral vein. An anastomosis of the proximal and distal part of the femoral artery was also performed.

**Figure 2:**
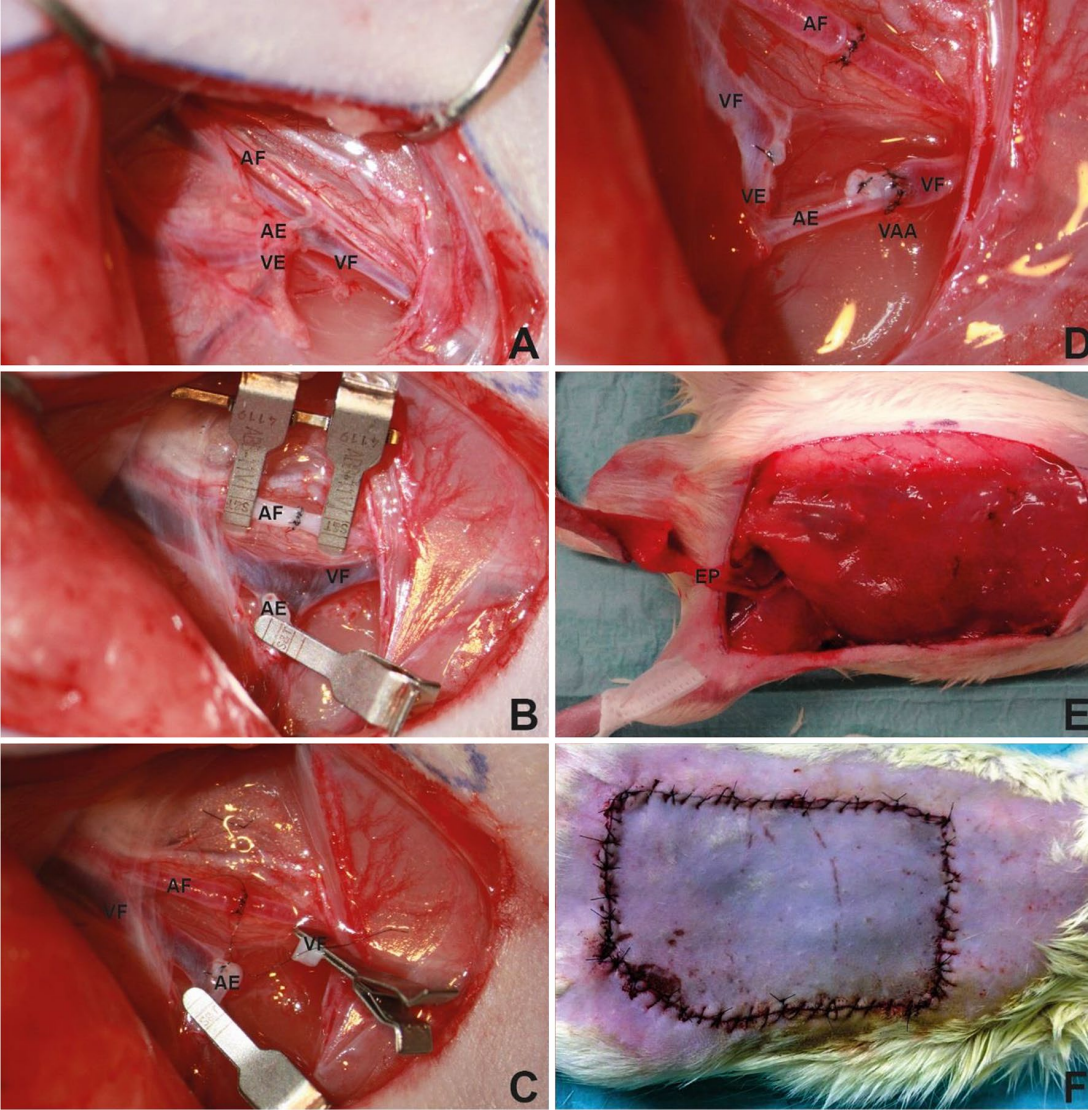
Clinical situation of the surgical procedure. (**A**) Femoral artery and vein with the epigastric branches. (**B**) Dissected epigastric vessels with its femoral origin and re-anastomosed femoral artery. (**C**) The re-perfused femoral artery, ligated branch of the proximal femoral vein, and first stitch in the distal femoral vein. (**D**) Anastomosis between the distal femoral vein and the epigastric artery. The distal femoral vein shows an arterial red color that can be explained by the retrograde arterial inflow from the mammarian artery. (**E**) Raised flap that is now perfused only by the epigastric vessels. (**F**) Flap sutured back into the wound bed in group III (+ 14 d). The flap appears slightly livid compared with the surrounding tissue. AF—arteria femoralis; AE—arteria epigastrica; EP—epigastric pedicle; VF—vena femoralis; VE—vena epigastrica; VAA—veno-arterial anastomosis.

Access to the epigastric vessels was obtained via a 3–4 cm long left inguinal incision. After preparation and isolation of the femoral vessels, the side branches were ligated, and the epigastric vessels were dissected 1–2 cm distal to the femoral root. In preparation for anastomosis, the femoral vein and artery were ligated distal to the epigastric vein and proximal to the epigastric artery, respectively. The epigastric artery, along with a short segment of the distal femoral artery, was anastomosed end-to-end with the distal portion of the femoral vein. The continuity of the femoral artery was reestablished by anastomosing the proximal and distal portions of the femoral artery (Figs. 1 and 2).

### Experimental groups

All rats were randomized by a computer-generated list and subdivided into four groups (*n* = 11 each), comprising three experimental groups (I–III) and one control group (IV). Following a strict protocol, a different technique was performed for each group (Table 1). Group I included rats with a veno-arterial anastomosis without preconditioning: the epigastric flap was raised, and a purely venous perfusion system was constructed. On the same day, the flap was sutured back into the epigastric defect (Fig. 1).

**Table 1:** Experimental protocol for the different groups (*n* = 11 rats each group). Groups I–III included rats with a veno-arterial anastomosis. Group I included rats with a veno-arterial anastomosis without preconditioning: the epigastric flap was raised, and a purely venous perfusion system was constructed. On the same day, the flap was sutured back into the epigastric defect (Fig. 1). In group II, a veno-arterial anastomosis was constructed, but the flap was raised and set back into the epigastric defect 7 days later for preconditioning. In group III, a similar venous-only perfusion was constructed, but the flap was raised and set back after an interval of 14 days for a longer preconditioning period. In the control group (IV), a standard arterial epigastric flap (based on the epigastric pedicle) was raised, the epigastric artery with its femoral origin was cut and re-anastomosed, and the flap was directly sutured back into the epigastric defect. All flaps were assessed 7 days after flap raising.

Group #	Day 0	Day 7	Day 14	Day 21
I (+ 0 d)	Veno-arterial anastomosis and flap raising	Euthanasia and flap assessment		
II (+ 7 d)	Veno-arterial anastomosis	Flap raising	Euthanasia and flap assessment	
III (+ 14 d)	Veno-arterial anastomosis		Flap raising	Euthanasia and flap assessment
IV–controlgroup (+ 0 d)	Arterial anastomosis and flap raising	Euthanasia and flap assessment		

In group II, a veno-arterial anastomosis was constructed, but the flap was raised and set back into the epigastric defect 7 days later to realize preconditioning. In group III, a similar venous-only perfusion was constructed, but the flap was raised and set back after an interval of 14 days for a longer preconditioning period.

In the control group (IV), a standard arterial epigastric flap (based on the epigastric pedicle) was raised, the epigastric artery with its femoral origin was cut and re-anastomosed, and the flap was directly sutured back into the epigastric defect.

All flaps were assessed 7 days after the flap was raised (Table 1).

### Indocyanine green (ICG) videoangiography

In addition to observing flap color, capillary refill, blood loss, and palpation of the anastomosis, ICG videoangiography was performed to describe fluorescence behavior and blood flow direction in the flaps and critically assess the patency of the microvascular anastomoses. Accordingly, we injected ICG (ICG-PULSION, Pulsion Medical Systems AG; Munich, Germany) intravenously and used the integrated fluorescence imaging device of the microscope (OPMI^®^ Pentero^®^, Carl Zeiss AG; Oberkochen, Germany). The imaging device was centered perpendicularly over the surgical field with a standardized distance of 30 cm. To assess the anastomosis and the flap, ICG videoangiography was performed for each experiment after each surgical step (veno-arterial anastomosis, flap raising, and flap assessment). First, an angiography of the flap, followed by another angiography of the anastomosis, was performed. The ICG (0.3 mg/kg body weight, 25 mg dissolved in 5 mL of sterile water) was injected intravenously as a bolus into the central venous catheter. The fluorescence signal was visualized on the video screen in real time (25 images/second) and recorded immediately after dye injection for 80 s, as described previously^29^. The mean ICG intensity of the flap was analyzed, as described previously^29^.

Morphometric angiographic analysis of the vascular network was performed using a method described by Takeshita et al.^41^. A composite of 5 mm^2^ grids was placed over the flap area. The total number of grid intersections and intersections crossed by an ICG-stained vessel were counted individually by an observer blinded to the treatment regimen. An ICG vessel score was calculated for each ICG angiography, as the ratio of grid intersections crossed by vessels divided by the total number of grid intersections in the flap^29^. Furthermore, the time from injection to the first ICG signal in the flap was recorded.

### Planimetric measurement of necrotic areas

Seven days after flap raising and setting it back, all rats were put under anesthesia, and flap healing was documented using a digital SLR camera (Nikon Coolpix 8700, Nikon Corp., Chiyoda, Tokyo, Japan) mounted perpendicular to the flap with a tripod. Vital and necrotic areas in the images were analyzed. Thus, the total flap area and necrotic areas were manually circumscribed using a graphic tablet, and the cross-sectional area was calculated using NIH Image Software (Image J 1.41; National Institutes of Health; Bethesda, MD, USA)^30^.

### Statistics

All observations were independently evaluated by two investigators blinded to the experimental groups. For basic statistical analysis, Prism 7 for Mac OS X Version 7.0a (GraphPad Software, Inc., La Jolla, CA, USA) was used. The IBM SPSS Statistics for Windows version 22 (IBM Corp., Armonk, NY, USA) was used for the multivariate analysis. The necrosis rate and mean ICG intensity of the flap were assessed using ordinary one-way analysis of variance (ANOVA) to determine significant between-group differences. Mean values and standard deviation (SD) were used to describe the flooding speed of the ICG signal in the flap. The time from ICG injection to its detection in the flap and vascular network was assessed using ordinary one-way ANOVA to determine significant between-group differences. Tukey’s multiple comparison testing was used to account for the problem of multiple testing. Differences were considered statistically significant for a two-sided *p* value of < 0.05. All data are presented as mean ± SD.

## Results

A total of 51 rats underwent surgery. Seven rats could not be included because of autodigestion or adverse events of anesthesia. The corresponding experimental series was conducted with 11 animals per group. In total, 44 rats survived the postoperative period and tolerated the anesthesia and operative procedures. In the postoperative monitoring of the flaps, a more livid color of the venously perfused epigastric flaps was observed in groups II and III, especially in comparison with the normal color of the non-transplanted skin (Fig. 2F). Flap edema was not observed in groups II and III.

### Flap survival

**Figure 3:**
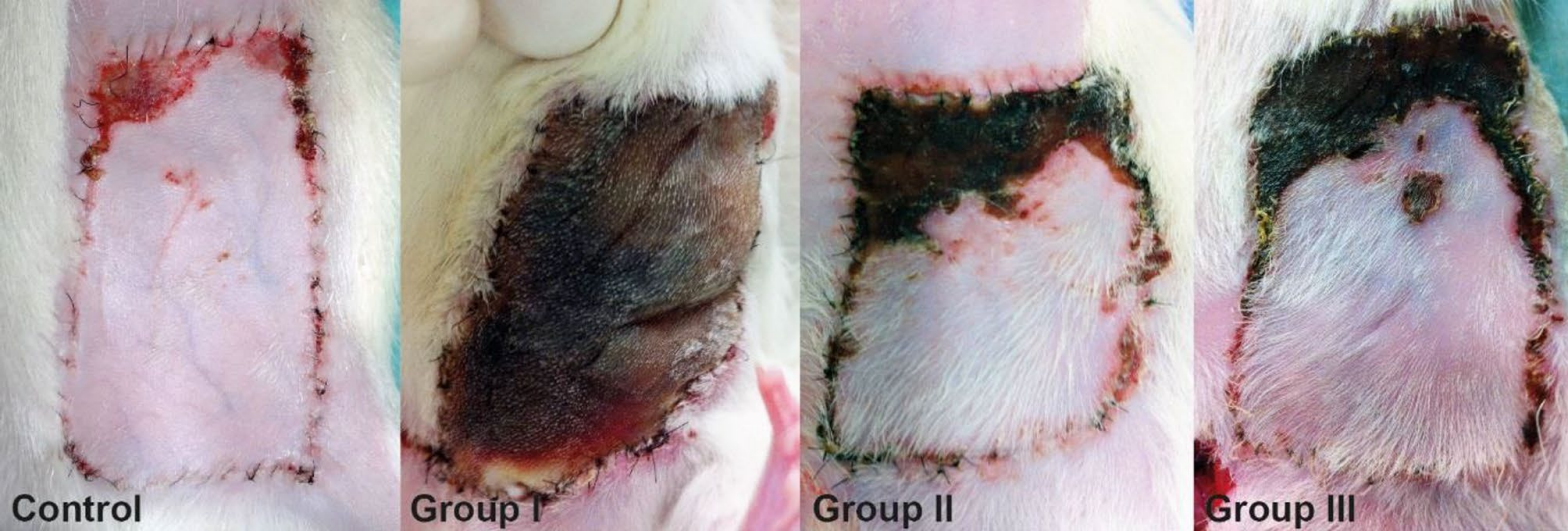
Flap survival. The mean survival rate in the control group was 89.3% ± 6.51%, total flap necrosis was found in group I, the mean survival rate in group II was 49.97% ± 24.34%, and that in group III was 64.95% ± 20.36%. In all groups, a necrosis pattern developed from mediocranial to laterocaudal, which corresponds with the findings of the perfusion pattern in ICG videoangiography (Fig. 4I).

In the comparison of the different groups, obvious and significant differences were found in the resulting necrosis rates (Fig. 3). The control group had a vitality rate of 89.3% ± 6.51%, group I had 0%, group II had 49.97% ± 24.34%, and group III had 64.95% ± 20.36%. A significant difference was found between the control group and groups I (*p* < 0.01), II (*p* < 0.01), and III (*p* < 0.01) and between groups I and II (*p* < 0.01). Significant differences were also found between groups I and III (*p* < 0.01) and between groups II and III (*p* = 0.034).

### ICG videoangiography

**Figure 4:**
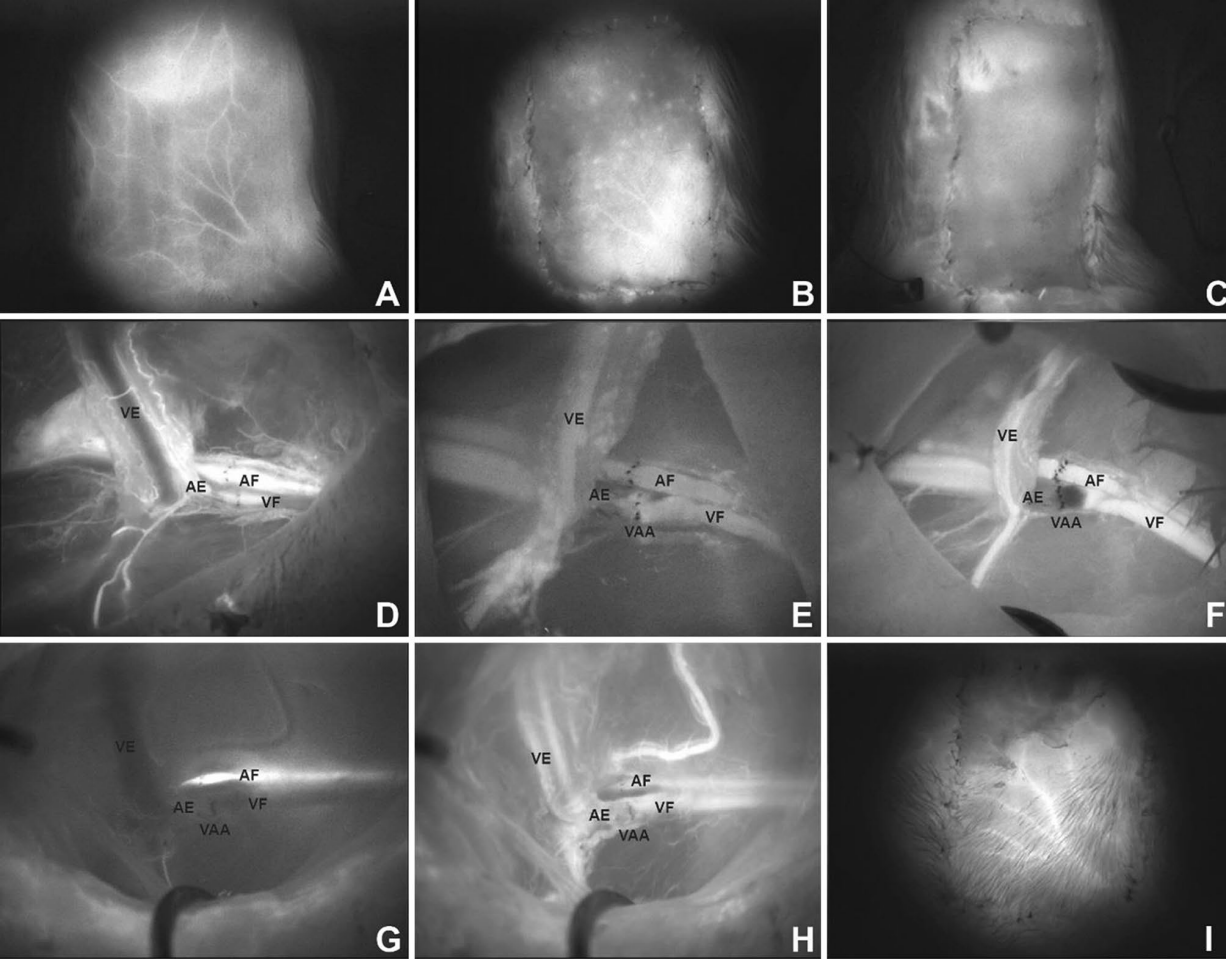
Results of ICG videoangiography. Decreased perfusion after flap raising (B, venous phase at 60 s) compared with flap perfusion before the raising [(**A**) arterial phase at 15 s)] in groups II and III. Note the perfusion pattern after flap raising (**B**); no ICG fluorescence in the flap immediately after flap raising in group I [(**C**), venous phase at 75 s]; venous flow-through the anastomosis in group I was not seen [(**F**) venous phase at 65 s]; venous perfusion through the anastomosis in groups II and III 7 days after flap raising [(**H**) venous phase at 40 s] clearly increased compared with that directly after raising [(**E**) venous phase at 57 s]; no retrograde perfusion at the end of the experiment [(**G**) arterial phase at 15 s] compared with retrograde arterialized femoral vein through mammarian artery directly after microanastomosis [(**D**) arterial phase at 17 s]; increased ICG fluorescence at the end of the experiment [(**I**), venous phase at 45 s] compared with the perfusion pattern directly after flap raising [(**B**) venous phase at 60 s] in groups II and III. AF—arteria femoralis; AE—arteria epigastrica; VF—vena femoralis; VE—vena epigastrica; VAA—veno-arterial anastomosis.

In all rats with veno-arterial anastomosis, ICG videoangiography revealed a reversed blood flow after the anastomosis, with blood supply derived from the internal mammary artery. In detail, the newly established blood flow ran anterogradely from the internal mammary artery to the anastomosis in the groin region and subsequently arterialized the distal femoral vein, and this resulted in vein dilation. Flap raising and corresponding transection of all perforators from the internal mammary and lateral thoracic artery resulted in a pure venous supply of the epigastric flap because the retrograde arterial perfusion of the femoral vein could no longer be ascertained (Fig. 4G). In groups II and III (7-/14-day interval), a slight ICG fluorescence intensity distal to the anastomosis was registered, whereas in group I (raising and setting back immediately after the anastomosis), fluorescence intensity could barely be detected (Fig. 4D/E/F). The ICG videoangiography of the flap perfusion immediately after flap harvesting showed a much stronger fluorescence intensity in groups II and III than in group I, where no recognizable ICG fluorescence was determined (Fig. 4A/B/C). The ICG fluorescence spread from laterocaudal to mediocranial, whereby the craniomedial parts were less perfused.

Groups II and III showed a more pronounced ICG perfusion of the flaps at the end of the experiment compared with flap perfusion immediately after flap raising (Fig. 4B/I). In the examination of the vessels, the perfusion of the venous anastomosis improved compared with the state just after flap raising (Fig. 4E/F/H).

After the experiment, the venous perfusion through the anastomosis in groups II and III 7 days after flap raising clearly increased compared with that directly after raising, and no retrograde perfusion was found (Fig. 4E/H/G). The ICG fluorescence still spread from laterocaudal to mediocranial, whereby the craniomedial parts were also still less perfused and showed corresponding areas of necrosis. However, this observation could not be analyzed statistically because no corresponding objective parameters were chosen for this purpose.

Regarding the mean intensity analysis in group I, no flap perfusion was detected as all cases had complete necroses clinically. A significant difference was detected in the mean intensity immediately after flap raising (*p* < 0.01), but not after the reevaluation of the epigastric flaps after 7 days between groups II and III (156.45 AU vs. 173.65 AU; *p* = 0.089). However, in the final angiography, the ICG signal in the flap was detected faster in group III than in group II (8.33 ± 1.21 s vs. 15.50 ± 3.45 s; *p* < 0.01). In addition, the vascular network, described as the ratio of grid intersections crossed by vessels divided by the total number of grid intersections in the flap, in group III was generally more precisely visible than the level in group II (0.50 AU ± 0.08 versus 0.32 AU ± 0.05; *p* < 0.01) (Fig. 4).

## Discussion

Venous flaps differ from conventional microvascular free flaps (nutrition via an arterial inflow). Normally, venous flaps are perfused completely by the venous system, including a venous inflow and outflow. In the present model, the inflow was maintained, but the arterial side of the vessels was replaced by the inflow of venous blood. In this rat model, oversized flaps were raised to evaluate the differences between each experimental group. The timing of vascular conditioning is an important step in the overall survival of these flaps. Additionally, the duration of flap conditioning appears to be a crucial factor for flap survival. Fluorescence angiography immediately after flap raising showed significantly better ICG intensities in group III than in group II. Although there was no higher ICG intensity between-group II and III at the end of the trial, clinically, significantly better flap survival with longer conditioning was observed group III than in group II. The flow-in time of ICG in the flap was faster in group III than in group II, as well as a higher density of the capillary network, which also appears to be a positive effect of longer conditioning.

The effect can be postulated due to an initially better perfusion of the flaps, and a longer conditioning is reasonable. In this study, soft tissue flaps with pure venous blood supply can be sufficiently perfused. Previously, venous tissue flaps were mainly applied in finger or toe reconstruction^7,20,21^. These defects are usually relatively small, and venous flaps are selected for the reconstruction of functionally important regions in which split-skin or whole-skin grafts could cause too much scarring and severely impair functional outcomes^7,20,21^. Some authors refer to venous flaps as “venous skin grafts”^31^ because the epithelial part of the flap mostly undergoes necrosis. In our experiment, we could not confirm this observation because the epithelium was not necrotic in the well perfused area (Figs. 3 and 4). Yoshimura et al.^31^ anastomosed distal veins of the flap with arteries to increase the oxygenation within the flap, which was successful in 12 of 13 cases; however, all flaps had partial necroses. Thatte et al. described purely venous perfused flaps, which led to survival rates of 0–30%^32^, whereas arterialized venous flaps showed survival rates of approximately 75%, comparable to group III in this study^33,34^. Although arterialized venous flaps have shown the best outcomes to date, the present technique showed similar results, containing a new aspect in the use of venous tissue grafts when no arterial connection is present. This effect could represent a new possibility for the use of venous soft tissue flaps. The conditioning of the vascular system and flap could be performed in a short preparatory surgery before the actual reconstruction. This advantageous conditioning step could take place in the first operative step because many clinics perform a secondary reconstruction (two-staged approach) after appropriate tumor ablation, when the histological examination of a resected tumor is performed^35,36^. The qualitative results of intraoperative ICG videoangiography in the present study showed that the immediate transposition of the flap’s vascular supply into a venous system without preconditioning did not lead to sufficient flap perfusion. Generally, adequate flap perfusion and continuous blood flow-through the anastomoses were observed in the fluorescence angiographic examination in the two-staged procedures of groups II and III. Further, increased vascularization in both preconditioned flap groups was seen in ICG videoangiography, represented by a higher density of the vascular network. Considering the clinical results as indicated by the survival rates, the circulation within the flap was assumed to adapt to the blood pressure of the venous system on the arterial side of the flap. Interestingly, no outflow problems occurred, which may be caused by the lower venous inflow and may serve as a starting point for further investigations. Moreover, the vascular network was angiographically well perfused after establishing blood flow over 14 days (group III) compared with the vascularization after 7 days (group II) and immediately after the creation of the veno-arterial anastomosis (group I). The present mechanism is suggestive of “hemodynamic remodeling” because of the perfusion change and adaptation over time. In contrast to arterialized venous flaps such as those described in the literature, this remodeling type turns the high-pressure arterial perfusion into a low-pressure one, which appears to be more physiological. The new veno-arterial blood flow is constant and unidirectional physiologically^37^. These effects show the physiological remodeling of the arterial vasculature adopted to the venous constant and low-oxygenated blood flow^38^. Similar to other attempts^8,13,25,39,40^, this study showed that there is much potential in the conditioning of transplants, although the physiological basis is still not understood comprehensively. Whether the ischemic conditioning of the transplant or the arterial retrograde vascular conditioning is responsible for the survival rate should be investigated.

Furthermore, whether retrograde perfusion in the sense of a veno-venous flap with appropriate conditioning leads to equal, better, or worse flap survival should be explored. Thus, whether nutrient and oxygen supply are secondary in doubt and the vascular system and corresponding hemodynamic remodeling are not primarily decisive for flap survival could be further evaluated. The extent to which this experimental form of free flaps could represent a real alternative in clinical use must be investigated in follow-up experiments, especially since venous connecting vessels are often lacking in critical conditions.

## Conclusion

In our rat epigastric flap model, free flaps with pure venous blood supply can be sufficiently perfused. The timing of vascular conditioning proved to be an important step in the overall survival of these flaps. Additionally, the duration of flap conditioning appears to be a crucial factor for flap survival. Whether ischemic conditioning of the transplant or arterial retrograde vascular conditioning is responsible for the survival rate remains to be investigated.

## Data Availability

The data that support the findings of this study are available from the corresponding author upon reasonable request.
